# Anti-oxidant and anti-microbial properties of some ethno-therapeutically important medicinal plants of Indian Himalayan Region

**DOI:** 10.1007/s13205-016-0470-2

**Published:** 2016-07-18

**Authors:** Sandeep Rawat, Arun K. Jugran, Amit Bahukhandi, Asutosh Bahuguna, Indra D. Bhatt, Ranbeer S. Rawal, Uppeandra Dhar

**Affiliations:** 1 G. B. Pant Institute of Himalayan Environment and Development, Kosi-Katarmal, Almora, Uttarakhand 263643 India; 2 Biotechnology Division, CSIR-Institute of Himalayan Bioresource Technology, Palampur, Himachal Pradesh 176061 India; 3 G. B. Pant Institute of Himalayan Environment and Development, Garhwal Unit, Srinagar, Uttarakhand 246174 India; 4 Department of Biotechnology and Microbiology, Modern Institute of Technology, Dhalwala, Rishikesh, Uttarakhand India; 5 Guru Gobind Singh Indraprastha University, Dwarka, New Delhi, 110078 India

**Keywords:** Anti-microbial agent, Ant-ioxidant, Medicinal plants, Phenolic content, Himalaya, In vitro assay

## Abstract

Therapeutic potential of medicinal plants as a source of noble natural anti-oxidants and anti-microbial agents has been well recognised all across the globe. In this study, phenolic compounds, in vitro anti-oxidant activity and anti-microbial properties have been investigated in five Himalayan medicinal plants, (e.g., *Acorus calamus*, *Habenaria intermedia*, *Hedychium spicatum*, *Roscoea procera* and *Valeriana jatamansi*) in different solvent systems. *R. procera* exhibited significantly (*p* < 0.05) higher phenolics; while *H. spicatum* was rich in flavonoids and *V. jatamansi* in anti-oxidant activity. Also, *R. procera* and *H. spicatum* were found rich in gallic acid; *V. jatamansi* in catechin, hydroxylbenzoic acid and caffeic acid and *H. intermedia* in hydroxyl benzoic acid. Solvent systems showed species specific response for extraction of total flavonoids and anti-oxidant activity. All the extracts were found effective against different bacterial and fungal strains in a dose dependent manner and maximum antimicrobial activity was found in *R. procera *as compared to other species. All the plant extracts showed greater activity against bacterial strains as compared to fungal strains. The results of this study suggest that extract of these species can be used as natural anti-oxidant to reduce free radical mediated disorders and as natural alternative for food preservation.

## Introduction

The therapeutic potential of medicinal plants as a source of noble natural anti-oxidants and anti-microbial agents has been well recognized world-wide (Cowan 1999; Aqil and Ahmad 2003; Vasinauskiene et al. 2006; Tenore et al. 2011). This potential might be due to the presence of several biomolecules i.e., phenolics, flavonoids, alkaloids, terpenoids, etc., which have distinct mechanism of action (Ajaib et al. 2011). Among these, phenolics and flavonoids are recognised for their multiple biological effects including anti-oxidants, anti-inflammatory, anti-microbial, anti-carcinogenic properties, etc. (Rice-Evans et al. 1997; Cowan 1999). As such, anti-oxidants are known to prevent various diseases e.g., diabetes, coronary artery diseases, cancer, inflammatory diseases etc., by decreasing localized oxygen concentration; preventing chain initiation by scavenging radicals; decomposing lipid peroxides to peroxyl and alkoxyl radicals; decomposing peroxides by converting them to non radical products, and chain breaking to prevent continued hydrogen abstraction (Deshpande et al. 1996; Bhatt et al. 2013). Similarly, anti-microbial property of medicinal plants is preventive against various infectious diseases and degradation of food products during production, storage and processing. A number of reports are available on the anti-oxidants (Wojdylo et al. 2007; Ajaib et al. 2011; Rawat et al. 2011) and anti-microbial properties of medicinal plants (Vlietinck et al. 1995; Essawi and Srour 2000; Vasinauskiene et al. 2006; Joshi et al. 2008). However, in case of many Himalayan medicinal plants, no such detail studies are available. Therefore, there is the need of systematic evaluation of these medicinal plants. Keeping all these facts in view, this study is performed to evaluate anti-oxidant and anti-microbial properties of five ethno-therapeutically important medicinal plants namely *Acorus calmus,* *Habenaria intermedia,* *Hedychium spicatum*, *Roscoea procera*, and *Valeriana jatamansi* using different solvent systems. All these species are used traditionally for treating various ailments (Table 1). Besides, *R. procera* and *H. intermedia* are known for their vitality strengthening properties and used as ingredients of Chyavanprash (a herbal combination used in traditional Indian Medicinal System), which is known to protect degenerative diseases and maintain youthfulness, vigor, vitality, etc. (Bhatt et al. 2013). The rhizomes of *H. spicatum* are stomachic carminative, stimulant and tonic, and used for curing dyspepsia, asthma and bronchitis, and used as a poultice for various acnes and pains due to its anti-microbial properties (Joshi et al. 2008; Rawat et al. 2011). The essential oil of *A. calamus* and *V. jatamansi* is used in mental/nervous disorders and several other purposes (Raina et al. 2003; Kalim et al. 2010). Thus, most of the species are used in food/herbal formulations, it become crucial to analyze the anti-oxidant activity and anti-microbial properties of these species (Table 1).

**Table 1:** Details of traditional medicinal uses and active content of selected medicinal plants

Plant species	Family	Traditional uses	Active constituents	References
*Acorus calamus*	Aracacerae	Asthma, epilepsy, chronic diarrhea, dysentery, bronchial catarrh and abdominal tumors, hysteria, syncope and mental weakness	β-asarone α-asarone, caryophylene, isoasarone, methyle isoeugenol and safrol	Raina et al. (2003), Phongpaichit et al. (2005)
*Habenaria intermedia*	Orchidaceae	Burning sensation, fever, cough, asthma, leprosy and skin diseases. Tuber is edible and used as depurative, anthelmentic, rejuvenating and tonic	Unknown	Warrier et al. (1994), Prajapati et al. (2003)
*Hedychium spicatum*	Zingiberaceae	Dyspepsia, nausea, asthma, vomiting, pain and inflammation. Boiled rhizome is edible and seeds are eaten with lentils	Hedychenone, spicatanol and 6-hydroxy-cineol	Joshi et al. (2008), Rawat et al. (2011)
*Roscoea procera*	Zingiberaceae	Impotency, diabetes, leucorrhoea, diarrhea, dysentery, and malaria. Tubers are edible and used in preparation of rejuvenating and tonic	Unknown	Rawat et al. (2014)
*Valeriana jatamansi*	Valerianaceae	Obesity, skin disorder, insanity, epilepsy and snake poisoning	Valepotriates, valeric acids and flavonoids	Jugran et al. (2013), Kalim et al. (2010)

Among the target species, rhizome extract and essential oil of *H. spicatum* have showed inhibitory activity against pathogenic bacterial and fungal strain including a strain of methicelin and vaneomycim resistant *Streptococcus aureus* (Joshi et al. 2008; Bisht et al. 2006). *V. jatamansi* essential oil exhibited anti-microbial activity against large number of pathogenic bacteria and showed potent anti-fungal activity against different human and plant fungal pathogens (Girgune et al. 1980; Sati et al. 2011). The anti-fungal activity of crude methanolic extract of *A. calamus* is reported (Ghosh 2006; Singh et al. 2010), and the essential oil of *A. calmus* exhibited anti-bacterial activity against phyto-pathogenic bacteria (Vasinauskiene et al. 2006). Also, anti-microbial activities of the crude methanol extract of rhizome and leaf extract of *A. calamus* is known (Phongpaichit et al. 2005; Asha-Devi and Ganjewala 2009).

Keeping the above in view, this study is attempted to (1) screen the phenolic and flavonoid content and anti-oxidant activity in selected medicinal plants using different solvent systems, (2) quantify various phenolic compounds using HPLC; and (3) evaluate anti-microbial activity against various pathogenic bacterial and fungal strains.

## Materials and methods

### Plant material

The plant material of selected species, i.e., *H. spicatum* (rhizome), *R.* *procera* (rhizome), *V. jatamansi* (roots), *H. intermedia* (tubers) and *A. calamus* (rhizome) were collected from wild populations in Uttarakhand (West Himalaya), India, in flowering phase (March–September 2009). The plant specimens were authenticated and deposited in the herbarium of G.B. Pant Institute of Himalayan Environment and Development, Kosi-Katarmal, Almora. The plant material collected from each species was dried separately in hot air oven at 50 °C for 10–12 days and dried material was grounded into fine powder using Willey grinder mill (Micro Scientific, New Delhi, India). Powdered material was kept in dry, cool and dark place prior to use for the quantification of phenols, flavonoids and evaluation of anti-oxidant and anti-microbial activity.

### Extract preparation

Plant material (1.0 g) of each species was extracted in five different solvents, i.e., water, methanol, ethanol, acetone and hexane, and heated in a water bath (60 °C) for 2 h and finally kept for 24 h incubation with continuous stirring at room temperature. Supernatant was filtered and dried using rotary evaporator (Strike-12, Steroglass, Italy). The extract was re-dissolved in methanol for the analysis of total phenolics, flavonoids, anti-oxidant and anti-microbial activity.

### Estimation of total phenolic and flavonoid content

Total phenolics were measured using standard methodology with minor modifications (Rawat et al. 2011). Briefly, a small portion (0.25 ml) of the methanolic extract described above was transferred into a test-tube containing 2.25 ml of distilled water, followed by addition of 0.25 ml of Folin–Ciocalteu’s reagent and allowed to stand for reaction for 5 min at 22 ± 1 °C,. The mixture was neutralized by adding 2.50 ml of 7 % (w/v) sodium carbonate. Than kept in the dark at 22 ± 1 °C for 90 min. The absorbance of the resulting blue-color solution was measured at 765 nm using a Hitachi U-2001 spectrophotometer (Tokyo, Japan). Quantification was based on a standard curve of gallic acid prepared in 80 % (v/v) methanol and the results were expressed in mg gallic acid equivalents (GAE)/g dry weight (dw) of plant material.

Total flavonoid concentrations in the methanolic extract of each sample was determined by the aluminium chloride colorimetric method with minor modifications (Bhatt et al. 2012). Briefly, the extract (0.50 ml) was diluted with 1.50 ml of distilled water, followed by the addition of 0.50 ml of 10 % (w/v) aluminium chloride, 0.10 ml of 1.0 M potassium acetate and 2.80 ml of distilled water. The mixture was incubated at 22 ± 1 °C for 30 min and its absorbance was recorded at 415 nm. Quantification was carried out on the basis of standard curve of quercetin prepared in 80 % (v/v) methanol and the data were expressed in mg quercetin equivalents (QE)/g dw plant material.

### Determination of anti-oxidant capacity

The anti-oxidant activity of the extracts of target species was analyzed in five different solvents using three different in vitro assays i.e., ABTS (2,2-Azinobis-3-ethylbenzothiazoline-6-sulphonic acid), DPPH (1,1-Diphenyl-2-picrylhydrazyl radical), and FRAP (Ferric reducing anti-oxidant potential) assays (Rawat et al. 2011; Bhatt et al. 2012).

### HPLC analysis of individual phenolic compounds

High performance liquid chromatography (HPLC) was used to measure the composition and concentrations of phenolic compounds in the samples of each species. The HPLC system (Merck-Hitachi, Tokyo, Japan) was equipped with an L-7100 series pump connected to an L-7400 series UV–VIS detector fitted with Winchrome 99 software (Infotech Instrument, Mumbai, India). Phenolic compounds in the extracts were separated in a Lichrosphere 100 RP-8e column (250 mm × 4.6 mm i.d, 5 µm pore size; Merck Pvt. Ltd, Tokyo, Japan) using on 80:20:1 (v/v/v) water:methanol:acetic acid mix as the mobile phase at a flow rate of 0.8 ml min^−1^ in the isocratic mode. Spectra of gallic acid, catechin, 3-hydroxybenzoic acid, *p*-coumaric acid, and ellagic acid were recorded at 254 nm, and those of caffeic acid and chlorogenic acid were recorded at 370 nm. The identification of individual phenolic compounds was based on their retention times compared to external standards (Sigma-Aldrich, St. Louis, MO, USA). UV–VIS spectra of the pure standards at different concentrations were used to plot calibration curves for the quantification of each phenolic compound. The reproducibility of the standards during the quantitative analysis was <3.0 % (intra-day relative standard deviation) for each phenolic compound. The results were expressed in mg/100 g dw of plant material.

### Determination of anti-microbial activity

#### Test microorganism

Bacterial and fungal strains were obtained from Microbial Type culture collection (MTCC), Institute of Microbial Technology, Chandigarh (India). Selection of fungal and bacterial strains were made on the basis of their pathogenic nature, wider availability, ability of antibiotic resistance and popularity. The bacterial strains included three Gram-positive strains namely, *Bacillus subtilis* (MTCC 441), *Staphylococcus aureus* (MTCC 196) and *Micrococcus luteus* (MTCC 106) and one Gram-negative strain namely, *Escherichia coli* (MTCC 739). The fungal strain included *Aspergillus flavus* (MTCC 1973), *Aspergillus fumigatus* (MTCC 3070), *Microsporum gypseum* (MTCC 2829) and *Candida albicans* (MTCC 1637). The bacteria were grown in the nutrient broth at 37 °C and maintained on nutrient agar slants at 4 °C. Fungal culture was grown in Sabouraud’s Dextrose broth at 28 °C and maintained on Sabouraud’s Dextrose Agar (SDA) (Hi-Media Pvt. Ltd, Mumbai, India) at 4 °C.

#### Anti-microbial activity screening

The anti-microbial activity of each extract was measured at five different concentrations (0, 50, 100, 250 and 500 mg/ml). Each experiment was performed in triplicate and a negative control containing methanol without plant extract was used. The anti-microbial activity was measured using agar disc diffusion method with disc size of 5 mm (Himedia, Delhi, India) (Natarajan et al. 2005; Bisht et al. 2006; Ghosh 2006). The sterile nutrient agar was inoculated with overnight grown microbial culture (200 μl of microbial cell suspension in 20 ml agar medium) and poured into sterile Petri dishes. Sterile filter paper discs of 5 mm diameter were impregnated with 20 μl of the equal concentration of extract solution. Thus, each paper disc absorbed doses of 0, 1, 2, 5 and 10 mg plant material, respectively for 0, 50, 100, 250 and 500 mg/ml concentration. The paper discs were dried and placed on the surface of the inoculated agar plates. Plates were kept for 2 h in refrigerator to enable pre-diffusion of the extracts into the agar and incubated for 48 h. Cell density was adjusted with sterile saline to 10^8^ cells/ml using spectrophotometer for inoculation of agar plates. An aliquot (200 μl) of inoculum was added to Molten Muller Hinton Agar No. 2 (Himedia, Delhi, India). Vancomycin, ciprofloxacin, ampicillin and streptomycin were used as positive control against bacterial strains and ketoconazole and fluconazole against fungal strains. Incubation period is followed by the evaluation of inhibition zones (diameter of inhibition zone plus diameter of the disc) for determining anti-microbial activity.

### Statistical analysis

All determinations of total phenols, flavonoids, phenolic compounds and anti-oxidant activity (using different assays) were conducted in five replicates along with separate extraction. Values for each sample were calculated as mean ± standard deviation (SD) and were subjected to analysis of variance (ANOVA). Significant differences among mean values were tested using Duncan’s multiple range test (DMRT; *p* ≤ 0.05) using SPSS software Version 17.0 (SPSS Inc, Chicago, IL). Correlation coefficients (*R*) and coefficients of determination (*R* ^2^) were calculated using Microsoft Excel 2007.

## Results

### Total phenolic and flavonoid content

Total phenolic content varied significantly (*p* < 0.05) among the studied species and maximum value (mean of all solvents) observed for *R. procera* (19.10 mg GAE/g dw) followed by *V. jatamansi* (12.82 mg GAE/g dw), *A. calamus* (7.35 mg), *H. intermedia* (3.39 mg) and *H. spicatum* (2.74 mg) (Table 2). While, comparing different solvents, the phenolic content was observed maximum in methanol extract (26.99 mg GAE/g dw) of *R. procera* followed by acetone (26.21 mg), ethanol (22.44 mg), aqueous (17.61 mg) and hexane (2.23 mg) extract, while, in *V. jatamansi* phenolic content was maximum in methanolic extract (14.37 mg GAE/g dw) followed by hexane (13.80 mg), ethanol (12.76 mg), acetone (12.30 mg) and aqueous extract (10.86 mg). Acetone was observed appropriate solvent for extraction of phenolic content in *A. calamus* and *H. spicatum*. Total flavonoid content was observed maximum (mean of all solvents) in *H. spicatum* (6.85 mg QE/g dw) and minimum in *R. procera* (5.05 mg). Total flavonoids content in *H. spicatum* was found maximum in hexane (7.58 mg QE/g dw) followed by ethanol (7.42 mg), methanol (6.57 mg), acetone (5.82 mg) and aqueous extract (4.76 mg). For extraction of total phenolic content, methanol (*H. intermedia, R. procera* and *V. jatamansi*) and acetone (*A. calamus* and *H. spicatum*) were found to be the best among all studied solvents. However, in case of total flavonoid content, different solvents performed differently among the species. For example, hexane was found best solvent for *H. spicatum*, ethanol for *R. procera* and *H. intermedia*, acetone for *V. jatamansi* and methanol for *A. calamus* (Table 2). Factorial analysis indicated that there were a significant (*p* < 0.01) effect of solvents on the extraction of total phenolics and flavonoids and significant variation (*p* < 0.01) among the species. Also, significant relation between species and solvents revealed that different solvents performed differently in these species for extraction of total phenolic and flavonoids content (Table 3).

**Table 2:** Total phenolic, flavonoids content and anti-oxidant activity in selected medicinal plants extract extracted in different solvents

Species	Total phenolic content (mg/g GAE dw)	Total flavonoid content (mg/g QE dw)	Anti-oxidant activity (AAE/100 g dw)		
			ABTS	DPPH	FRAP
*Acorus calamus*					
Aqueous	6.56 ± 0.22	4.14 ± 0.05	12.49 ± 0.34	4.15 ± 0.08	1.62 ± 0.06
Methanol	7.93 ± 0.03	5.96 ± 0.06	27.51 ± 0.19	6.66 ± 0.07	4.72 ± 0.04
Ethanol	6.97 ± 0.03	5.30 ± 0.06	26.52 ± 0.20	5.55 ± 0.11	4.13 ± 0.05
Acetone	8.01 ± 0.09	5.55 ± 0.07	23.47 ± 0.36	6.30 ± 0.08	4.25 ± 0.04
Hexane	7.27 ± 0.11	4.37 ± 0.09	26.40 ± 0.27	5.40 ± 0.06	4.63 ± 0.02
Average	7.35	5.06	23.28	5.61	3.87
*F-value *	84.99**	408.39**	1456.88**	457.73**	2463.95**
LSD (*p* < 0.01)	0.30	0.17	0.24	0.2	0.12
*Habenaria intermedia*					
Aqueous	2.11 ± 0.15	4.81 ± 0.03	6.73 ± 0.09	5.25 ± 0.09	6.71 ± 0.07
Methanol	4.77 ± 0.10	6.56 ± 0.00	6.74 ± 0.10	8.58 ± 0.01	12.05 ± 0.13
Ethanol	4.01 ± 0.15	9.54 ± 0.03	9.89 ± 0.09	8.49 ± 0.35	11.84 ± 0.22
Acetone	3.49 ± 0.09	4.97 ± 0.01	6.30 ± 0.08	8.50 ± 0.04	10.83 ± 0.07
Hexane	2.55 ± 0.09	7.04 ± 0.02	7.72 ± 0.27	8.27 ± 0.65	11.51 ± 0.04
Average	3.39	6.58	7.48	7.82	10.59
*F-value *	257.01**	18735.77**	312.14**	56.85**	967.86**
LSD (*p* < 0.01)	0.30	0.63	0.37	0.86	0.32
*Hedychium spicatum*					
Aqueous	2.55 ± 0.04	4.76 ± 0.01	6.55 ± 0.17	8.34 ± 0.43	2.26 ± 0.36
Methanol	3.27 ± 0.02	6.57 ± 0.01	9.77 ± 0.13	12.21 ± 0.22	10.93 ± 0.24
Ethanol	2.85 ± 0.07	7.42 ± 0.01	7.56 ± 0.27	11.85 ± 0.08	10.01 ± 0.18
Acetone	3.36 ± 0.05	5.82 ± 0.01	6.46 ± 0.36	11.20 ± 0.42	6.49 ± 0.35
Hexane	1.65 ± 0.03	7.58 ± 0.01	6.75 ± 0.09	7.14 ± 0.81	10.66 ± 0.22
Average	2.74	6.85	7.42	10.15	8.07
*F-value *	32.02**	38785.39**	111.91**	72.12**	516.78**
LSD (*p* < 0.01)	0.12	0.27	0.59	1.2	0.73
*Roscoea procera*					
Aqueous	17.61 ± 0.15	5.07 ± 0.02	14.10 ± 0.39	13.33 ± 0.48	3.35 ± 0.20
Methanol	26.99 ± 0.08	5.73 ± 0.02	16.39 ± 0.07	13.88 ± 0.01	18.13 ± 0.27
Ethanol	22.44 ± 0.14	6.16 ± 0.02	15.68 ± 0.22	12.86 ± 0.02	12.34 ± 0.15
Acetone	26.21 ± 0.05	5.68 ± 0.01	12.95 ± 0.08	12.65 ± 0.07	12.49 ± 0.32
Hexane	2.23 ± 0.12	2.61 ± 0.01	9.44 ± 0.06	7.92 ± 0.04	3.68 ± 0.28
Average	19.10	5.05	13.71	12.13	10.00
*F-value *	23607.82**	3376.13**	1005.77**	366.74**	1927.71**
LSD (*p* < 0.01)	0.30	0.44	0.54	0.56	0.65
*Valeriana jatamansi*					
Aqueous	10.86 ± 0.04	5.47 ± 0.01	60.62 ± 1.83	9.82 ± 0.08	4.67 ± 0.05
Methanol	14.37 ± 0.59	5.58 ± 0.02	95.81 ± 0.28	10.88 ± 0.07	6.47 ± 0.29
Ethanol	12.76 ± 0.04	6.15 ± 0.01	85.35 ± 0.20	10.36 ± 0.14	5.80 ± 0.17
Acetone	12.30 ± 0.07	6.24 ± 0.01	68.60 ± 2.34	10.07 ± 0.12	6.70 ± 0.20
Hexane	13.80 ± 0.16	5.36 ± 0.02	75.47 ± 0.27	10.22 ± 0.07	5.97 ± 0.27
Average	12.82	5.76	77.17	10.27	5.92
*F-value *	71.11**	2436.23**	320.13**	47.17**	40.79**
LSD (*p* < 0.01)	0.71	0.37	0.35	0.26	0.55
Among species					
LSD (*p* < 0.01)	0.15	0.33	0.62	0.27	0.20

*GAE* gallic acid equivalent, *QE* quercetin equivalent, *AAE* ascorbic acid equivalent, *ABTS* 2,2-azinobis-3-ethylbenzthiazoline-6-sulphonic acid, *DPPH* 1,1-diphenyl-2-picrylhydrazyl assay, *FRAP* ferric reducing anti-oxidant potential assay, *LSD* least significant difference

Level of significance: ** significant at (*p* < 0.01)

**Table 3:** Analysis of variance for total phenolic, flavonoid content and anti-oxidant activity in selected medicinal plants

Source of variance	*df*	Total phenolic content		Total flavonoids content		ABTS		DPPH		FRAP	
		Ms	*F*-value	Ms	*F*-value	Ms	*F*-value	Ms	*F*-value	Ms	*F*-value
Solvent (*S*)	4	712.4	29,939.3**	5.49	4789.1**	12,906.4	32,303.9**	120.27	1553.1**	95.95	2333.6**
Plant species (*P*)	4	85.8	3606.3**	8.61	7511.6**	284.1	711.1**	12.17	157.2**	99.19	2412.7**
*S* × *P*	16	58.4	2457.5**	2.45	2139.8**	105.7	264.6**	3.38	43.6**	26.90	654.4**
Error	50	0.02		0.00		0.40		0.08		0.04	
Total	74	856.62		16.55		13,296.6		135.9		222.08	

*df* Degree of freedom, *Ms* mean square

Level of significance: ** significant at (*p* < 0.01)

### Anti-oxidant activity

Anti-oxidant activity measured by three in vitro assays varied among the species (Table 2) and the maximum activity irrespective of the solvent was observed in case of *V. jatamansi* (mean value = 77.17 mM AAE/100 g dw), followed by *A. calamus* (23.28 mM), *R. procera* (13.71 mM), *H. intermedia* (7.48 mM) and *H. spicatum* (7.42 mM) using ABTS assay. Anti-oxidant activity using DPPH showed the maximum activity in *R. procera* (12.13 mM AAE/100 g dw) and minimum in *A. calamus* (5.61 mM). FRAP assay showed maximum activity in *H. intermedia* (10.59 mM AAE/100 g dw) and minimum in *A. calamus* (3.87 mM). Anti-oxidant activity measured by three in vitro assays showed a significant variation (*p* < 0.01) across solvent systems as well as among species (Table 2). Among the solvents, methanol showed significantly higher (*p* < 0.01) anti-oxidant activity measured by all three assays (i.e., ABTS, DPPH and FRAP) in all the species except *V. jatamansi* (FRAP assay) and *H. intermedia* (ABTS) where acetone and ethanol, respectively exhibited highest anti-oxidant activity (Table 2). Factorial analysis revealed a significant (*p* < 0.01) effect of solvent system on anti-oxidant activity (Table 3). Also, interaction of solvents and plant species also revealed significant effect on the anti-oxidant activity (Table 3). While correlating the phenolic and flavonoid content with anti-oxidant activity, DPPH assay showed a highly significant positive relationship (*p* < 0.001) with total phenolic content. Also, FRAP assay showed a significant relationship (*p* < 0.05) with total flavonoid content (Table 4).

**Table 4:** Correlation matrix of total phenolic content, flavonoids and anti-oxidant activity measured by three in vitro assays

	Total phenolic content	Total flavonoid content	ABTS	DPPH	FRAP
Total phenolic content	1				
Total flavonoids content	−0.067	1			
ABTS	0.329	−0.052	1		
DPPH	0.618***	0.245	0.141	1	
FRAP	0.320	0.561*	−0.267	0.508*	1

Level of significance: * *p* < 0.05, *** *p* < 0.001

### Phenolic compounds

Six phenolic compounds namely, gallic acid, catechin, hydroxyl benzoic acid, caffeic acid, chlorogenic acid and *p*-coumaric acid were identified and quantified through HPLC (Table 5). Gallic acid content ranged between 2.48 mg/100 g (*H. intermedia*) and 56.94 mg/100 g (*R. procera*). Similarly, catechin ranged between 5.12 mg/100 g (*R. procera*) and 85.87 mg/100 g (*V. jatamansi*), however, it was not detected in *H. intermedia.* Hydroxyl benzoic acid ranged from 3.90 mg/100 g (*H. spicatum*) to 57.22 mg/100 g (*V. jatamansi*), and it was not detected in *A. calamus* and *R. procera.* Caffeic acid was only detected in *A. calamus* (3.25 mg/100 g) and *V. jatamansi* (55.81 mg/100 g) while, chlorogenic acid was only detected in *V. jatamansi* (4.41 mg/100 g) roots. *p*-coumaric acid was found maximum in *V. jatamansi* (1.47 mg/100 g) followed by *H. spicatum* (0.81 mg/100 g) and *R. procera* (0.52 mg/100 g) and it was not detected in *A. calamus* and *H. intermedia*. Comparative analysis among the species revealed that *R. procera* exhibits maximum gallic acid (56.94 mg/100 g dw), while other phenolic compounds such as, catechin (85.87 mg/100 g), hydroxyl benzoic acid (57.22 mg/100 g) and caffeic acid (55.81 mg/100 g) were maximum in *V. jatamansi*.

**Table 5:** Phenolic compounds (mg/100 g dw) in selected medicinal plants extract extracted in 80 % methanol

Species name	Gallic acid	Catechin	Hydroxyl benzoic acid	Caffeic acid	Chlorogenic acid	Coumaric acid
*Acorus calamus*	13.94 ± 0.24	5.12 ± 0.31	nd	3.25 ± 0.16	nd	nd
*Habenaria intermedia*	2.48 ± 2.77	nd	18.51 ± 2.02	nd	nd	nd
*Hedychium spicatum*	19.76 ± 0.75	7.47 ± 0.05	3.90 ± 0.12	nd	nd	0.81 ± 0.06
*Roscoea procera*	56.94 ± 5.25	6.50 ± 0.39	nd	nd	nd	0.52 ± 0.03
*Valeriana jatamansi*	9.39 ± 1.39	85.87 ± 4.28	57.22 ± 7.96	55.81 ± 8.17	4.41 ± 0.31	1.47 ± 0.15

*Nd* not detected

### Anti-microbial activity

Results obtained from disc diffusion method for anti-microbial activity of the plant extracts in different solvents against eight pathogenic micro-organisms are shown (Table 6). All the plant extract exhibited strong anti-bacterial activity. Results indicated that ethanolic extract of *R. procera* was found most effective against all bacterial strains in different solvents in varying concentrations. For instance, the extract of *R. procera* was active against *Escherichia coli,* *Bacillus subtitis* and *Staphylococcus aureus* in all the doses (1, 2, 5 and 10 mg) but was active against *Micrococcus luteus* only in high dose (2, 5 and 10 mg). Ethanolic extract of *V. jatamansi* showed maximum activity against all studied bacterial strains except *B. subtilis*, which was found sensitive to acetone extract of the species. *H. intermedia* and *V. jatamansi* extracts in all the solvents were also found to inhibit the growth of *E. coli*. Whereas, *A. calamus* and *H. spicatum* extractions did not inhibit the growth of *S. aureus* in any concentration. Also, hexane extract of *A. calamus* and *H. spicatum* did not inhibit the growth of *B. subtilis*. Ethanolic extract of *H. intermedia* exhibited maximum activity against *E. coli*, *B. subtilis* and *S. aureus* while, *M. luteus* remained most sensitive against the acetone extract. This suggests that the quality of active compounds responsible for anti-microbial activity varies in different extracts (Table 6).

**Table 6:** Antimicrobial activity revealed by inhibition zone diameters (mm) of plant material extracted in various solvents in different doses (mg/disc) of selected medicinal plants against various pathogenic bacterial and fungal strains

Name of plant species	Solvent type	Zone of inhibition (mm) in different doses (mg/disc)															
		*B. subtilis*				*S. aureus*				*M. luteus*				*E. coli*			
		1	2	5	10	1	2	5	10	1	2	5	10	1	2	5	10
A. Anti-bacterial properties																	
*Acorus calamus*	Aqueous	–	–	–	–	–	–	–	–	–	–	–	6	9	6	–	7
	Methanol	8	–	–	–	–	–	–	–	–	–	–	8	–	–	7	–
	Ethanol	6	6	8	10	–	–	–	–	–	–	–	7	6	6	6	9
	Acetone	–	–	–	9	–	–	–	–	–	–	6	7	6	–	7	9
	Hexane	–	–	–	–	–	–	–	–	–	6	6	9	–	–	–	–
*Habenaria intermedia*	Aqueous	–	–	–	–	–	–	–	7	–	–	6	8	6	6	7	–
	Methanol	–	–	–	–	–	–	6	8	–	–	–	–	6	–	6	6
	Ethanol	7	6	6	11	–	–	6	9	–	–	6	6	7	6	8	11
	Acetone	–	6	6	11	–	–	6	9	–	6	7	9	6	–	8	7
	Hexane	–	–	–	–	–	–	–	–	–	–	–	7	–	–	8	–
*Hedychium spicatum*	Aqueous	6	6	–	–	–	–	–	–	6	6	6	8	–	6	7	–
	Methanol	–	–	–	–	–	–	–	–	6	6	6	–	–	–	7	9
	Ethanol	7	6	–	–	–	–	–	–	6	8	9	6	–	–	6	7
	Acetone	–	–	–	–	–	–	–	–	6	6	–	–	–	–	–	–
	Hexane	–	–	–	–	–	–	–	–	–	–	7	–	–	–	–	–
*Roscoea procera*	Aqueous	7	6	6	–	7	6	–	–	–	6	6	6	7	6	8	10
	Methanol	8	6	7	–	–	6	7	7	–	–	7	7	7	6	7	6
	Ethanol	7	7	8	8	6	–	7	7	–	6	9	9	8	7	10	12
	Acetone	–	9	8	8	6	8	10	10	–	6	6	6	7	7	7	7
	Hexane	–	6	6	–	–	–	–	–	–	–	7	7	–	6	7	6
*Valeriana jatamansi*	Aqueous	–	–	7	7	–	–	–	–	–	–	7	6	–	–	7	6
	Methanol	–	8	–	8	–	6	–	–	–	–	6	7	8	–	7	6
	Ethanol	–	–	6	8	6	7	6	6	–	6	9	6	6	6	6	8
	Acetone	–	9	11	11	7	–	6	8	–	6	8	7	7	–	9	7
	Hexane	–	–	8	7	–	–	–	–	–	6	7	6	–	–	6	–
Vanomycin (30 μg/disc)		20				18				35				22			
Ciproflaxacin (10 μg/disc)		35				23				33				31			
Streptomycin (10 μg/disc)		22				25				19				20			
Ampicillin (10 μg/disc)		10				09				13				33			

Name of plant species	Solvent type	Zone of inhibition (mm) in different doses (mg/disc)															
		*A. flavus*				*A. fumigatus*				*M. gypseum*				*C. albicans*			
		1*	2	5	10	1	2	5	10	1	2	5	10	1	2	5	10
B. Anti-fungal properties																	
*Acorus calamus*	Aqueous	–	–	–	–	–	–	–	–	–	–	–	–	–	–	–	–
	Methanol	–	–	–	–	–	–	–	–	–	–	–	–	–	–	–	7
	Ethanol	–	–	–	–	–	–	–	–	–	–	–	–	–	–	6	6
	Acetone	–	–	6	8	–	–	–	–	–	–	–	–	–	6	7	9
	Hexane	–	–	–	–	–	–	–	–	–	–	–	–	–	6	6	6
*Habenaria intermedia*	Aqueous	–	–	–	–	–	6	–	–	–	–	–	–	6	6	–	6
	Methanol	–	–	–	–	–	–	–	–	–	–	–	–	6	6	–	6
	Ethanol	–	–	–	–	–	–	–	–	–	–	–	–	6	–	–	6
	Acetone	–	–	–	–	–	–	–	–	–	–	–	–	6	7	–	9
	Hexane	–	–	–	6	–	–	6	6	–	–	–	–	6	6	–	6
*Hedychium spicatum*	Aqueous	–	–	–	–	–	–	–	–	–	–	–	–	–	–	–	–
	Methanol	–	–	–	–	–	–	–	–	–	–	–	–	6	–	–	–
	Ethanol	–	–	–	–	–	–	–	–	–	–	–	–	6	–	–	–
	Acetone	–	–	–	–	–	–	–	–	–	–	–	–	6	6	6	–
	Hexane	–	–	6	6	–	–	–	–	–	–	–	–	–	6	7	7
*Roscoea procera*	Aqueous	–	–	–	–	–	–	–	–	–	–	–	–	6	6	6	6
	Methanol	–	–	–	–	–	–	–	–	–	–	–	–	7	6	6	6
	Ethanol	–	–	–	–	–	–	–	–	–	–	–	–	6	6	–	–
	Acetone	–	–	–	–	–	–	–	–	–	–	–	–	6	7	6	–
	Hexane	–	–	–	–	–	–	–	–	–	–	–	–	7	6	7	7
*Valeriana jatamansi*	Aqueous	–	–	–	–	–	–	–	–	–	–	–	–	6	6	6	–
	Methanol	–	–	–		6	6	–	–	–	–	–	–	–	6	6	6
	Ethanol	–	–	–	6	–	–	–	–	–	–	–	–	6	6	6	6
	Acetone	–	–	–	–	–	–	–	–	–	–	–	–	–	6	6	8
	Hexane	–	–	–	–	–	6	6	–	–	–	–	–	6	–	7	7
Ketoconazole (10 μg/disc)					20				14				10				18
Fluconazole (10 μg/disc)					18				17				12				22

– No activity shown; values are represented as mean of three determinations; * dose of plant material (mg/disc)

Among the fungal strains, sensitivity was recorded only against three fungal strains (i.e., *A. flavus*, *A. fumigatus* and *C. albicans*) and none of the plant showed activity against fungal strain *M. gypseum*. *A. fumigates* did not exhibit sensitivity against *H. spicatum*, *R. procera* and *A. calamus* extracts. Likewise*, A. flavus* did not show sensitivity against *R. procera* (Table 6). All the plant extracts in different solvent systems showed activity against *C. albicans* except aqueous extract of *H. spicatum.* Similarly, *A. flavus* showed sensitivity against hexane extract of *H. spicatum* and *H. intermedia*. *A. fumigatus* was found sensitive against hexane extract of *H. intermedia* and *V. jatamansi* at high concentration. It was also revealing that all the plant extracts showed greater activity against bacterial strains as compared to fungal strains.

## Discussion

This is a systematic investigation on anti-oxidant and anti-microbial potential of selected edible and ethno-therapeutically important medicinal plants of west Himalaya (i.e., *H. spicatum*, *R. procera*, *H. intermedia*, *V. jatamansi* and *A. calamus),* which justify their role in food preservative and reported their ethno-therapeutic uses. Although reports on anti-oxidant activity in some of these species are available but the anti-microbial activity of *R. procera* and *H. intermedia* against bacterial and fungal strain is reported for the first time. Similarly, extract of *H. spicatum* was tested first time against some pathogenic bacterial strains, such as, *B. subtitis* and *M. luteus*. While, the study on anti-oxidant activity of *H. intermedia* is first of its kind.

Among all the selected species, total phenolic and flavonoid content were relatively higher in *R. procera* extract, which suggested its comparative advantage over other species for anti-oxidant rich formulations. *R. procera* is used in preparation of anti-oxidant rich polyherbal formulation ‘Astavarga’ along with *H. intermedia* and 6 other medicinal herbs, which is well recognized for strengthening vital force of the body, cell regeneration capacity and immunity system (Rawat et al. 2014). However, previous report showed lower value of total phenolic content in *R. procera* (methanolic extract = 2.92 mg GAE/g dw; Rawat et al. 2014). This might be due to the difference in extraction methods and collection from different geographic locations with different genetic make-up. The process of heat incubation used in this study provides aglycosylation of bounded phenolic compounds into their free forms (Wojdylo et al. 2007). Phenolic and flavonoid content in *V. jatamansi* are in agreement with the previous findings, which showed phenolic content (12.79 mg GAE/g dw) and flavonoids (4.19 mg QE/g dw), in methanolic extract of wild individuals of the species (Bhatt et al. 2012; Jugran et al. 2013). However, this is contrary with the finding of Kalim et al. (2010) who reported higher values of total phenolic (73.13 mg GAE/g) and flavonoids content (74.32 mg QAE/g) in the 50 % methanolic extract of *V. jatamansi*. In case of *H. spicatum*, comparable total phenolic content (2.83–4.69 mg GAE/g) has been reported in methanolic extract of different samples collected from various geographic regions of Uttarakhand, in west Himalaya (Rawat et al. 2011). Likewise, a comparable value of total phenolic content (4.90 mg GAE/g dw) was observed in methanolic extract of *A. calamus* (Surveswaran et al. 2007; Bahukhandi et al. 2013). This study, therefore, indicates that most of the phenolic content present in the species are extractable in solvents with moderate polarity, such as, methanol, ethanol and acetone. Reports about total phenol and anti-oxidant activity in rhizome part of *A. calamus* are also available (Wojdylo et al. 2007) emphasizing that solvent plays a crucial role in extraction of phenolic compound due to their diverse and complex nature. Anti-oxidant activity of *H. spicatum* was found comparable using similar in vitro anti-oxidant assays reported earlier (Rawat et al. 2011), whereas, in case of *R. procera* it was comparatively lower with all the three assays (Rawat et al. 2014). Likewise, it was lower as compared to previous report on *V. jatamansi* using the ABTS assays (Bhatt et al. 2012; Jugran et al. 2013). Anti-oxidant activity (by DPPH assay) showed positive relationship with phenolic compounds and similar relationship between anti-oxidant activity and phenolic compounds have been reported in many plants from Himalayan origin (Rawat et al. 2011; Bhoyar et al. 2011; Bhatt et al. 2012; Badhani et al. 2015) or other than Himalayan origin (Wojdylo et al. 2007).

Phenolic and flavonoid compounds present in different solvent extract are capable of scavenging ABTS and DPPH radicals, and are able to reduce the ferric ions. Different solvent exhibited species specific response for the extraction of anti-oxidant activity and phenolic content. This may be due to that anti-oxidant activity is exhibited by an array of molecules having diverse polarities and solubility. Also, level of different phenolic compounds exhibited a great variation in occurrence of individual phenolic compounds in all the species, which leads to necessity of optimization of solvent for extraction. A strong positive correlation has been reported between phenolic content and DPPH and ABTS free radical scavenging activity and ferric ion reducing activity (FRAP) in various medicinal plants like *H. spicatum* (Rawat et al. 2011), *Capparis spinosa* (Bhoyar et al. 2011) and *Lafoensia pacari* (Sampaio et al. 2011). Generally, in phenolic compounds, the higher number of hydroxyl groups and those having hydroxyl groups in *ortho*-position in the aromatic ring usually quench more free radicals on molar basis. Among the quantified phenolic compounds, gallic acid (3,4,5-tri-hydroxy benzoic acid) with three hydroxyl group bonded to aromatic ring in the *ortho*-position possess more scavenging properties toward free radicals. Caffeic acid and catechin possess a similar *ortho*-dihydroxy moiety bonded to the aromatic ring in *ortho* (*o*–) position and also possess more affinity toward free radical. *Ortho* substitution (*o*–) of hydroxyl group (–OH) to the aromatic ring seems adequate for anti-oxidant and scavenging activity of phenolic acid. The lowest anti radical and anti-oxidant activity of some compounds having one hydroxyl group such as 3-hydroxy benzoic acid, 2-hydroxy benzoic acid (salicylic acid), *p*-coumeric acid (4-hydroxy cinnamic acid) exhibited the lowest anti-radical and anti-oxidant activity is due to the fact that anti-oxidant and anti-radical activity of phenolic acid has positively correlated with a number of hydroxyl groups to the aromatic ring (Sroka and Cisowski 2003).

Presence of anti-oxidant phenolic compounds ensures the role of these species against free radical generated diseases. Individually, gallic acid is polyhydroxyphenolic acid in nature and has been reported as free radical scavenger and induces apoptosis in leukemia, lung cancer, and colon adenocarcinoma cell lines, as well as in normal lymphocyte cells along with strong anti-mutagenic, anti-fungal, anti-bacterial and anti-viral activity (Sohi et al. 2003). Catechins are one of the powerful anti-oxidant from plant sources and known to reduce the risk of ischemic heart diseases by reducing oxidation of low density lipoproteins (Arts et al. 2001). Caffeic acid exhibited variety of potential pharmacological effects against in vitro and in vivo model systems. Recently, inhibitory effect of caffeic acid on cancer cell proliferation by oxidative mechanism in human HT-1080 fibrosarcoma cell line has been evaluated. It also shows immune-modulatory and anti-inflammatory activity and reducing aflatoxin production (Prasad et al. 2011). Chlorogenic acid exhibited anti-hypertensive effects and protective effect in neuro-inflammatory conditions on dopaminergic neurons (Shen et al. 2012). *p*-Coumaric acid has anti-oxidant properties and is believed to reduce the risk of stomach cancer by reducing the formation of carcinogenic nitrosamines (Kikugawa et al. 1983).

Besides anti-oxidant activity, phenolic compounds of these species may play a role in prevention of food spoilage thus can be utilized as food preservative agent. It has been reported that the rapid growth in antibiotic resistance and the increasing interest in natural products have found medicinal plants as a reliable source for the discovery of active anti-microbial agent and possibly even novel classes of antibiotics (Panghal et al. 2011). This becomes true when number of micro-organisms acquire several resistance mechanism; making them multi-drug resistance (MDR) and develop novel anti-microbial agent for combating resistant organism (Kenneth 2009). In this study, different bacterial and fungal strains exhibited variable sensitivity to various plant extracts at different concentrations while none of the extract was found effective against a fungus, *M. gypsaum*. A greater sensitivity of bacterial strains than fungal strain might be due to cellular complexity of organization of fungal strains over bacterial strains. All the plant extracts were found active against both Gram-positive and Gram-negative bacteria. This is contrary with the earlier reports, which suggest that plant extracts remain more active against Gram-positive bacteria as compared to Gram-negative bacteria (Perez et al. 1990; Vlietinck et al. 1995). While comparing with standard drugs e.g., Ampicillin, Vancomycin, Ciprofloxacin and Streptomycin, the plant extract demonstrated relatively lesser anti-microbial activity; however, the higher doses of the plant can be comparable. These results show potential utility of natural compounds over synthetic antibiotics considering the reported side effects of synthetic chemicals (Karaman et al. 2003). Also, the synthetic drugs have high production cost, therefore, biologically active compounds derived from plants can be an alternative source to combat infectious diseases (Essawi and Srour 2000). It was interesting to note that *R. procera* possess higher total phenolic content and anti-microbial activity. Generally, phenolic compounds serve as defensive mechanism against protection by micro-organisms, insects and herbivores in plants. The mechanism thought to be responsible for phenolic toxicity to micro-organism include enzyme inhibition by the oxidized compounds possible through reaction with sulfhydryl group or more non-specific interaction with proteins/enzymes inactivation (quinine, flavonoids and tannins); substrate deprivation (catechins), membrane disruption (epicatechin), binds to adhesins molecules preventing adhesion to host cell (epicatechin gallate, gallocatechin gallate, polyphenol) and complexed with bacterial cell walls, and making it permeable for other solutes etc. The site(s) and number of hydroxyl group are thought to be related to their relative toxicity to microorganism, with evidence that increased hydroxylation results in increased toxicity (Cowan 1999). Considering the anti-fungal and anti-microbial activity, all the species, can be considered as a natural preservative against food-borne pathogens for the food industry.

Hexane extract of *H. spicatum,* and *A. calamus* did not show sensitivity against *E. coli* in any concentration, while, methanolic extract of *V. jatamansi* was found much effective against bacterial strains. These results are an indicator that polar organic solvents exhibited greater anti-microbial activity and strengthening the fact that anti-microbial agents are polar in nature, therefore, can be extracted through polar organic solvents (Singh and Singh 2000; Natarajan et al. 2005). The results indicated the different sensitivity between Gram-positive and Gram-negative bacteria might be due to the morphological differences in the micro-organisms. Gram-negative bacteria having outer phospholipid membrane made up of lipo-polysaccharide components, thus making the cell membrane impermeable for lipophilic solutes (Nostro et al. 2000). Also, it is possible that lipophilic solutes extracted by hexane extract (non-polar solvent) are, therefore, not much effective against Gram-negative bacterium *E. coli*. Generally, Gram-positive were found comparatively more susceptible than Gram-negative bacteria, which might be due to the presence of outer peptidoglycan layer, which is not an effective permeability barrier (Nostro et al. 2000). However, in this study, more sensitivity of Gram-negative bacterial strains might be due to other possible mechanism of extracted constituents which are directly associated with membrane disruption, etc. (Cowan 1999).

This study provides first report on anti-microbial activity of *H. intermedia* and *R. procera.* The results of present investigation are largely in agreement with the earlier studies on anti-oxidant and anti-microbial activity of other species (Aqil and Ahmad 2003; Bisht et al. 2006; Asha-Devi and Ganjewala 2009; Sati et al. 2011; Rawat et al. 2011) and provide a lead towards searching new source of anti-oxidants and anti-microbial substances. As such, the medicinal plants have received much attention in the food and flavoring industries as a source of natural anti-oxidant and anti-microbial agents. In this context, the results of this study could prove valuable for promoting the use of such species as potential supplement of neutraceuticals. Also, the results obtained in this study prove the efficacy of traditional uses of the plants as herbal medicine and food preservatives. However, considering preliminary nature of these results, deeper investigation towards potential discovery of new natural bioactive compounds is essentially required. Also, investigation to establish synergy between anti-oxidant activity and anti-microbial activity is needed.

## Conclusion

Anti-microbial activity along with the anti-oxidant effectiveness of plant extracts is one of the most examined features, important for both food preservation and control of human and animal diseases of microbial origin. Numerous reports suggest strong anti-microbial and anti-oxidant activities of various plant extracts. Our results indicate that the most of the target species possess a good anti-oxidant activity and exhibit a broad spectrum of anti-microbial activity against referenced strains, especially bacterial strains. The results of this study suggest that extract of these species could be used as natural anti-oxidant to reduce free radical mediated disorders and may helpful as self preserving agent for its processed products and also may be a source of active molecule against disease causing pathogens.
